# Supplementary material for: Chromothripsis during telomere crisis is independent of NHEJ, and consistent with a replicative origin
Source: Genome Res. 2019 May;29(5):737–49. doi: 10.1101/gr.240705.118 (PMC6499312; doi:10.1101/gr.240705.118)
Supplement: Supplemental Material [file supp_gr.240705.118_Supplemental_file_1.zip › contigs/annotated_contigs/DB46/contig.2.DB46_length_711_mean_cov_4.57946554149.docx]

**DB46_length_711_mean_cov_4.57946554149**

```
CACAATGCCTCAAACAAACACTAAGTACAAATCACAATCAATTAGTAGTATTGAAAAAGACCTCTAAGAGTTATTGAACAAGTAATTTC
 >chr14:86048395-86048791 - E=2e-223 p=1e-02
TACCTCTTCCTTAGGGTCTCCATTCAAGCTTTCCGTCACTTTCACAGAGGTTCCTAAATTGAAACAAACCACAACTACACCCTGCATGC

ATATACAAACACATATATCATATAATCACAAGGTTGTATTTCATACGTATCAGAATAATGCTCATATGGCTACTCAACAGAATTCTATC

AACGCTCCATTTGTTTTTGCTTTACCATCAATCTACAGGATCTAAGAGCAGGATCTACATATGGATTCAACCTCCTATATCACACAGAT

TGTAGAGTGGTAGTTAACATTATGTAAGCACCTCCCA|TTG|GATTGGAATCAATAGAATGTCAAGATGCCAGGCACATTCAGCCTAAT
                                      >chr14:86046498-86046816 + E=2e-176
AGCAGGTGGGTGTCTGCTGGACAGTGTGCCTGGTTTGCCTGACAGTAGCAAATTTAGGCCAGAATTAATGAGCCAGGGAAGGTACTGTT

GGTGTTGCAAGATTTTTGCAGTTTTCAGAAGTAATTTTGTTTTTCCTTTTTAATGGGATTTTCAGAAGTTTTCAGAAGTAATTTTGTTT

CTCCTTTTTAATGGGATTACATTTGCAAAAGAAGTTTGTGTTCATATATGTGCGAGTGCATGCATGCCCCTTTATTATAATACCTTTAT

G
```
